# Tailored Prevention of Functional Decline through a Multicomponent Exercise Program in Hospitalized Oncogeriatric Patients: Study Protocol for a Randomized Clinical Trial^*^

**DOI:** 10.1007/s12603-023-1977-y

**Published:** 2023-10-05

**Authors:** Nicolas Martínez-Velilla, V. Arrazubi, F. Zambom-Ferraresi, I. Morilla-Ruiz, M.L. Sáez de Asteasuu, R. Ramírez-Vélez, F. Zambom-Ferraresi, A. De la Casa-Marín, I. Ollo-Martínez, I. Gorospe-García, I. Gurruchaga-Sotés, A. Galbete, B.A. Cedeño-Veloz, L. Martín-Nevado, M. Izquierdo, R. Vera

**Affiliations:** 1 Navarrabiomed, Hospital Universitario de Navarra (HUN)-Universidad Pública de Navarra (UPNA), IdiSNA, Pamplona, Spain; 2 CIBER of Frailty and Healthy Aging (CIBERFES), Instituto de Salud Carlos III, Madrid, Spain; 3 Geriatric Department, Hospital Universitario de Navarra (HUN), Pamplona, Spain; 4 Department of Medical Oncology, Hospital Universitario de Navarra, Pamplona, Spain; 5 Public University of Navarra, Pamplona, Spain; 6 Department of Geriatric Medicine, Hospital Universitario de Navarra, Irunlarrea 3, 31008, Pamplona, Spain

**Keywords:** Cancer, multicomponent exercise, functional capacity, disability, oncogeriatric, hospitalization

## Abstract

**Background:**

Cancer mostly affects older adults, causing a wide variety of diagnostic and therapeutic dilemmas. One of the most important moments in cancer patients is the hospitalization period, in which older patients usually remain bedridden for many hours and this may lead to the appearance of sarcopenia and disability.

**Methods:**

We present the research protocol for a randomized controlled trial that will analyze whether an intervention applied to older patients (≥ 65 years) who are hospitalized for acute medical conditions in an Oncology Department improves function. A total of 240 hospitalized older patients will be recruited in the Hospital Universitario de Navarra, Pamplona, Spain, and they will be randomized. The intervention consists of a multicomponent exercise training program that will take place for 4 consecutive days (2 sessions/day). The control group will receive usual hospital care, which will include physical rehabilitation when needed. The primary end point will be the change in functional capacity from baseline to hospital discharge, assessed with the Short Physical Performance Battery (SPPB). Secondary end points will be changes in cognitive and mood status, quality of life, fatigue, strength (dynamic and handgrip), pain, nutrition, length of stay, falls, readmission rate and mortality at 3 months after discharge.

**Results:**

Basal data of the patients included in the RCT are described. The foreseen recruitment will not be achieved due to the context of the Covid pandemic and the significantly different responses observed during the clinical trial in oncogeriatric patients compared to our previous experience in older adults hospitalized for medical reasons.

**Discussion:**

If our hypothesis is correct and shows that a multicomponent, individualized and progressive exercise program is an effective therapy for improving the capacity of acutely hospitalized older patients compared to usual care, a change in the current system of hospitalization may be justified in oncogeriatric patients.

## Abbreviations

ADLs: Activities of daily living
AE: Adverse events
HUN: Hospital Universitario de Navarra
EQ-5D: 6- EuroQol–5 Dimension questionnaire
GVT: Gait velocity test
KPS: Karnofsky Performance Status
MNA-SF: Mini-nutritional assessment- short form
RCT: Randomized clinical trials
RM: Repetition maximum
SPPB: Short Physical Performance Battery
TMT: Trail Making Test
VAS: Visual analog scales.

## Background

**T**he progressive ageing of society and the challenge posed by care for older adults has become a true urgency for all countries and health systems (1). One of the key points for the modification of these needs is the high prevalence of chronic diseases, including cancer. Furthermore, cancer mostly affects older adults, involving a wide variety of diagnostic and therapeutic dilemmas (2). In this context, the concept of vulnerability or frailty linked to the geriatric patient can be projected onto the cancer patient (3), where the role of sarcopenia is a particular determining factor (4, 5).

During the hospitalization period, it is very common the development of disability, which can irreversibly mark the life course of patients with cancer (6, 7). Almost 60% of patients remain bedridden during their stay for an average of 20 hours per day (8), results that can be extrapolated, at least partially, to cancer patients (9).

Although there is much evidence of the role of exercise in cancer patients (4, 5), a strategy related to the prevention of functional impairment in hospitalized oncogeriatric patients has not been fully developed. In recent years the number of studies investigating physical activity and exercise behavior of frail older adults is increasing (10). The European VIVIFRAIL physical exercise prescription model has been developed by experts worldwide in the field of physical activity and frailty (11, 12, 13, 14), and can be considered a milestone in this regard. Through a multicomponent exercise program, functional and cognitive impairment can be prevented even in hospital settings with very elderly patients and with a high burden of morbidity (11, 12, 13). Furthermore, the physiological mechanisms underlying the benefits of exercise in this population are not completely clear yet (14).

However, there are numerous nuances that make the implementation of similar models in hospitalized cancer patients a real challenge (15). Furthermore, the evidence for the management of geriatric cancer patients is limited by the scarcity of randomized clinical trials. Older adults are often excluded from early phase studies and randomized clinical trials (RCT) by restrictive inclusion criteria being only 1% of interventional trials older-patient-specific (16, 17).

Thus, the main aim of the present RCT will be to analyze the effects of short-term multicomponent exercise training on functional capacity outcomes in acutely hospitalized older patients with cancer.

## Methods/design

### Study Design and Setting

This study is a randomized clinical trial conducted in the Medical Oncology Department with patients older than 65 years of a tertiary hospital in Spain - Hospital Universitario de Navarra (HUN). The study follows the principles of the Declaration of Helsinki (18) and the protocol employs relevant standard items for clinical trials according to the SPIRIT 2013 statement (19) and follows the CONSORT statement (20) for transparent reporting. The trial is registered at ClinicalTrials. gov (NCT05424055), the status is recruiting, and the protocol was approved by the HUN Clinical Research Ethics Committee (PI_2020/7). All patients or their legal representatives provide written informed consent. Patient recruitment begin with the normal visit of the patient during hospitalization assessing for inclusion criteria and obtaining informed consent.

### Participants and eligibility

Patients are admitted to the hospital, to the Medical Oncology Department, regardless of the type of cancer, and usually in relation to complications related to their underlying disease or related treatments. Patients or their relatives (if the patient is unable to comprehend themselves) are informed of the random inclusion in one group but are not be informed which group the patient belongs to. The data for both the intervention group and the control group will be obtained at three different times: at baseline, before discharge, and at the 1-month followup.

The inclusion criteria are: age 65 years or older, Barthel Index score of 60 or more (scale, 0 [severe functional dependence] to 100 [functional independence]), being able to ambulate (with/without assistance), and to communicate and collaborate with the research team, and signing the informed consent form,. The exclusion criteria included are expected length of stay less than 6 days or more, terminal illness, very severe cognitive decline (ie, Global Deterioration Scale score, 7), uncontrolled arrhythmias, acute pulmonary embolism, acute myocardial infarction, and extremity bone fracture in the past 3 months. Inclusion and exclusion criteria are summarized in Table 1.

**Table 1:** Eligibility criteria

**Inclusion**	**Exclusion**
• Women and men aged ≥ 65 years	• Expected length of stay < 6 days
• Barthel Index ≥ 60 points	• Terminal illness
• Able to ambulate (with/without assistance)	• Very severe cognitive decline (i.e., GDS 7)
• Sign the informed consent	• Uncontrolled clinical situations as unstable infections, arrhythmias, acute pulmonary embolism, acute myocardial infarction or limb bone fracture in the past 3 months, or any clinical condition considered by the attending doctor as innapropriate for the trial.
• Able to communicate	


GDS: Global Deterioration Scale

After randomization, the research team (physiotherapist, sport science specialist, oncologist, and geriatrician) perform the baseline measurement and follow-up visits of functional, pharmacological, comorbidity, and cognitive assessment, as well as mobility and strength evaluations (Table 2). The multidisciplinary research team has previous experience in functional geriatric assessment and in the prescription of exercise in frail older patients in different clinical settings (12, 21). Regarding oncogeriatric patients, our previous findings confirm that a multicomponent exercise program improves measures of physical/cognitive functioning and quality of life in older adults with lung cancer under adjuvant therapy or palliative treatment, and some systematic reviews identify the best options and challenges in these type of patients (43, 44, 45, 46).

**Table 2:** Time of measurement of the different variables in each of the participants of the study

**Measurement**	**T1 Baseline**	**T2 After training or control period**	**T3 1-month post decharge**	**T4 3-months post decharge**
Categorical scale of pain	x	x	x	x
Handgrip strength	x	x	x	x
Barthel Index	x	x	x	x
Karnofsky's Index	x	x	x	x
Geriatric depression Scale of Yesavage (GDS)	x	x	x	x
Short Physical Performance Battery (SPPB)	x	x	x	x
Gait Velocity Test (GVT)	x	x	x	x
Dual-task (verbal and arithmetic GVT)	x	x	x	x
Maximal isometric force of handgrip	x	x	x	x
1RM (Leg press, Chest press and Knee extension)	x	x	x	x
Muscle power at 50 % 1RM in Leg press	x	x	x	x
Quality of Life (EQ-5D-5L)	x	x	x	x
EORTC QLQ-C30	x	x	x	x
Geriatric syndromes	x	x	x	x
Brief Fatigue Inventory	x	x	x	x
Trail Making Test part A (TMT-A)	x	x	x	x
Laboratory parameters	x	x		
Diseases considered grouped by ACG of Salisbury and CIE-10 codes	x			
Cumulative Illness Rating Scale for Geriatrics (CIRS-G)	x			
Falls	x	x	x	x
Mini Nutritional Assessment (MNA)	x	x		


1RM: 1 Repetition Maximum;

Basic sociodemographic and clinical data of the participants are collected in the baseline visit (Table 1). All adverse events, including those related exclusively to the exercise such as muscle pain, fatigue and general aches and pains, will be recorded in an “adverse events diary” during follow-up visits by the training and testing staff, and by self-report during the study period. The time at which different variables (primary and secondary) will be measured is detailed in Table 2. A blood sample will be taken on the days of the baseline and before discharge measurements.

The variables related to the tumor and the reason for, and evolution of admission will also be collected, distinguishing between toxicity due to chemotherapy (CTCAE v5.0), intercurrent medical complication, or neoplastic progression.

### Participant-Selection and Consent Process

Participants are identified during the oncologist assessment process at admission to the Oncology Department. If they agree, they will be forwarded to a space reserved for signing the free and informed consent form, an interview, and initial evaluation with the study researchers.

### Randomization, allocation concealment and Interventions blinding

**Figure 1:**
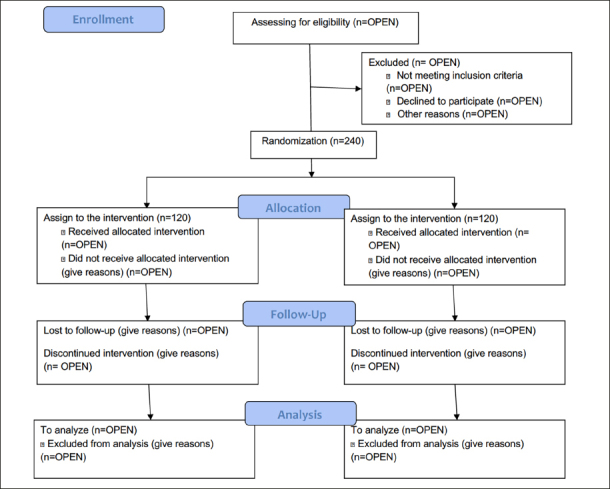
Flow diagram of the Study Protocol

Patients who meet the inclusion criteria are randomly assigned to the intervention or control group. Prior to randomization (http://www.randomizer.org), the attending oncologist review the absolute and relative contraindications to participating in the exercise program and will provide general information about the study. The allocation mechanism is carried out until 240 total patients are assigned. The assignment keys will be kept in a confidential file in the HUN and will be opened at the end of the study's analyses. To ensure masking, an alpha-numerical code will be assigned to each study group. This code is delivered to the associated researcher and is not revealed to the investigators in charge of processing the data until the analysis of the coded interventions is completed. Assessors of outcomes are blinded to patient data, including allocation at baseline and follow-up. Blinding is used for all laboratory analyses. Owing to the nature of the study, patients cannot be blinded to the exercise training modality. The study flow diagram is shown in Fig. 1.

All interventions take place at the Oncology Department of HUN (Pamplona, Spain) site facilities. Standard medical care are provided to all participants in all groups. Participants are randomly assigned to the following groups:

• Usual care group (control): Participants randomly assigned to the usual care group receive normal hospital care, including physical rehabilitation when needed.
• Multicomponent exercise group (intervention): The intervention consists of a multicomponent exercise training program which include supervised progressive resistance exercise training, balance-training and walking for 4 consecutive days. During the training period, patients are trained in 20-minute sessions twice a day (morning and evening).

**Figure 2:**
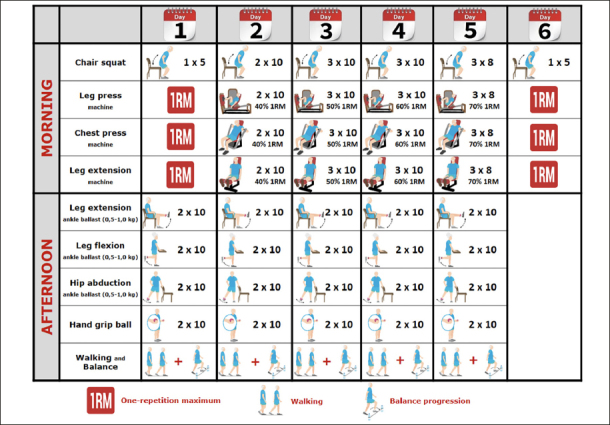
Intervention group exercise protocol

The supervised multicomponent exercise training program include upper and lower body strengthening exercises, tailored to the individual's muscle function, using weight machines and aiming for 2–3 sets of 8–10 repetitions at an intensity of 40–70 % of 1 repetition maximum (RM) (Matrix, Johnson Health Tech, Ibérica, S.L., Madrid, Spain) combined with balance and gait retraining exercises that will progress in difficulty and functional exercises, such as rises from a chair. The second part of the session consists of functional exercises such as knee extension and flexion, hip abduction, balance movements, and daily walking in the hospital. The resistance exercises are focused on the major upper and lower limb muscles. Each resistance training session includes two exercises for the leg extensor muscles (bilateral leg extension and bilateral knee extension muscles) and one exercise for the upper limbs (seated bench press). In each session, subjects perform a specific warm-up with one set of very light loads for the upper and lower body. The training protocol is shown in Figure 2. Participants and their family members are carefully familiarized with the training procedures in advance. During hospitalization, two evaluations are carried out; the first one taking place on the day of inclusion in the study and the second one on the 6th day after said inclusion.

One-Repetition Maximum (1RM).

### Outcome Assessment

The primary outcome is the change in functional status during the study period.

• The functional capacity of participants is evaluated by the Short Physical Performance Battery (SPPB) (22), with the total score ranging from 0 (worst) to 12 points (best). This test evaluates balance, gait and rising from a chair. The standing balance test consists of the evaluation of the ability to maintain the standing position for 10 seconds with three different feet-positions: parallel, semi-tandem and tandem. The walking speed test measures the time needed to progress for 4 linear meters at the patient's usual speed, assigning a different score according to speed. Finally, the chair sit-to-stand part assesses the ability to stand from a chair five consecutive times without using the arms. The SPPB test has been shown to be a valid instrument for screening frailty and predicting disability, institutionalization and mortality.

Secondary outcomes of the study will include:

• Handgrip strength. Handgrip strength is a useful tool for the measurement of functional capacity and predictor of disability (23). Each study participant will be asked to squeeze the dynamometer (Takei 5401 Digital Dynamometer) two different times and the higher score will be used to reflect handgrip strength. The measurement will be performed while the participant is seated, with the dominant hand with the elbow straight and the shoulder abducted at 15° (24, 25).
• Changes in functional capacity in activities of daily living (ADLs) as measured with the Barthel Index (Spanish version) scale, which ranges from 100 (functional independence) to 0 (severe functional dependence) (26). This is an international, validated tool and it is the most used to measure disability (27).
• Karnofsky' Index: The Karnofsky Performance Status (KPS) is a widely used method to assess the functional status of a patient.
• Gait ability will be assessed using the 6-meter gait velocity test (GVT). Starting and ending limits will be marked on the floor with tapelines for a total distance of 8 m. Participants will be instructed to walk at their self-selected usual pace for two attempts. The results of both trials will be averaged to obtain a single value. The first and last meters, considered the warm-up and the deceleration phases, respectively, will not be included in the calculations of the gait assessment.
• Dual-task GVT (arithmetic GVT). During the arithmetic dual-task arithmetic test (arithmetic GVT), we will assess the gait velocity while participants are counting backwards aloud from 100 one at a time. The cognitive score will be measured by determining how many numbers were counted backward (dual-task with arithmetic performance) (28).
• Changes in mood status: the 15-item Yesavage Geriatric Depression Scale (GDS-VE), the Spanish Version, contains ten affirmative and five negative items, its score being normal when it ranges from 0 to 5 and a score greater than 5 suggesting depression (29).
• Changes in the Trail Making Test (TMT) part A will be evaluated to assess executive dysfunction. The TMT is one of the most popular neuropsychological tests and is included in most test batteries. TMT-A requires an individual to draw lines sequentially connecting 25 encircled numbers distributed on a sheet of paper (30).
• Maximal dynamic strength will be assessed using the 1RM test in the bilateral leg press, knee extension and bench press exercises using exercise machines (Matrix, Johnson Health Tech, Ibérica, S.L., Madrid, Spain). In the first assessment, the subjects will warm up with specific movements for the exercise test. Each subject's maximal load will be determined in no more than five attempts, with a 3-minute recovery period between attempts. During all neuromuscular performance tests, strong verbal encouragement will be given to each subject to motivate them to perform each test action as optimally and rapidly as possible. Qualified fitness specialists will individually monitor and carefully supervise all training sessions and provide instruction and encouragement during all sessions.
• Changes in the quality of life will be measured by the Spanish version (31) of the EuroQol–5 Dimension (EQ-5D) questionnaire (32). It is an instrument that measures 5 dimensions of health status: mobility, self-care, usual activities, pain/discomfort, and anxiety/depression. Each dimension is rated according to the following levels: a) no problems; b) some problems; c) extreme problems. Besides, it contains a visual analogy scale to quantify the self-perceived health status, ranging from 0 (worst health state imaginable) to 100 (best health state imaginable).
• Number of days alive after admission to the hospital.
• Use of health resources: new admissions to the hospital, admission to nursing homes, visits to the emergency department and visits to the general practitioner.
• Visual analogue scales (VAS) typically ask a patient to mark a place on a scale that matches their level of pain (33);
• Mini-nutritional assessment- short form (MNA-SF): evaluation of nutritional status (34).
• Brief Fatigue Inventory: validated tools to measure fatigue in patients with cancer (35).

### Statistical procedures

The sample size calculation was performed using the GPower v. 3.1 software, adopting a significance level of α = 5%, a correlation between pre-and post-intervention values of the Short Physical Performance Battery (SPPB) of ϱ = 0.5 and a standard deviation for the SPPB of σ = 2.5, the required sample size to have a power of 90% to detect a minimum difference of 1 point between groups in the post–pre SPPB score is 101 patients per group. Taking an expected loss of patients at follow-up of 15% into account, the final sample size required is 120 per group.

The description of the sample by the group will be conducted using statistics such as means and standard deviations or medians and interquartile ranges for the quantitative variables, and frequencies and percentages for the qualitative variables. For comparisons between groups at baseline, t-tests or Mann–Whitney U tests will be used for continuous variables, depending on normality, which will be checked for each using the Kolmogorov-Smirnov test and normal probability plots, and the chi-square test or Fisher's test will be used for categorical variables.

To determine the efficacy of the intervention in the quantitative variables, such as the SPPB, we will use linear mixed models, using time, group and time and group interaction as categorical covariates. In the case of qualitative or categorized variables (such as whether an improvement of a given magnitude between pre-and post-intervention has been achieved or not), comparisons between groups will be conducted with the chi-square test or Fisher's test, and complemented with logistic regression if additional adjustment is needed. Effect sizes between-group will be computed based on Cohen's d and the 95% confidence interval. The level of statistical significance will be 0.05. Data will be analyzed using an intention-to-treat approach with R and SPSS statistical packages.

In order to check the assumption that the potential impact of missing data is negligible (created by a missing complete at random or a missing not at-random mechanism), best–worst and wors`t-best case sensitivity analyses will be used. When the missing data surpasses 5% and if this is supported by the previous sensitivity analysis, a missing at-random mechanism will be assumed and missing values will be imputed using the R package MICE (Multivariate Imputation via Chained Equations). If the proportion of missing data is very large (rises over 40%) on important variables, trial results will be considered hypothesis-generating results.

We will compare the proportion or number of adverse events between groups, using chi-square test or Mann-Whitney U test.

### Adverse events (AE) and safety evaluation

Safety is assessed by registering any adverse events suffered by the patients in the intervention group that could potentially be secondary to the physical exercise performed during the training sessions.

## Initial results

Patients show a mean age of 74.4 years (SD 5.3), mostly men (60%) and frail (Short Physical Performance Battery (SPPB)) around 8.5 (see supplement Table S1). This population is younger than in previous studies by our research group in geriatric wards, but at the same time shows a profile of frailty according to different variables like SPPB, hand grip, gait speed or functional measures.

## Discussion

Based on previous research showing the benefits of exercise in older adults admitted to the hospital because of medical reasons we have developed a RCT protocol for older patients with cancer. Given the relationship between both cancer and frailty (3), it makes sense that those interventions that are effective in frail patients could be beneficial in oncogeriatric patients and vice versa. This single-centre randomized controlled trial will be the first, to the best of our knowledge, to evaluate a short-term individualized multicomponent exercise program during the hospitalization period in oncogeriatric patients. A priori, the benefit could be even greater than that of the general population, but given the complexity and vulnerability of the elderly with cancer, the interventions may not have a similar response.

Although our previous research suggests that elderly patients should not be excluded from clinical trials based solely on their age, in the area of oncogeriatrics there is a wide margin of research on the effects of exercise during the hospitalization period. RCTs determine the standard of care treatment for older adults, yet they are often excluded from early phase studies. This highlights the role of geriatric oncology. Positive steps are being made, including the integration of geriatric assessment interventions for older patients with advanced cancer showing that this strategy can reduce the serious toxic effects of cancer treatment (38).

Most of the clinical evidence in oncology is based on pharmacological trials. In this study we propose the implementation of a new approach model for cancer patients, from a perspective that gets the focus of attention on function, giving it as much importance as the disease itself. As we have previously mentioned, the role of sarcopenia and muscle function is central in cancer patients, especially the elderly, therefore our intervention innovatively addresses cancer prevention. Ultimately, therapeutic priorities are diluted in geriatric patients, prioritizing quality of life over survival expectancy on many occasions (39). Proactive evaluation of functional status and appropriate interventions have been proposed by guidelines in oncogeriatric patients (40). Our intervention has not been tested among geriatric oncology patients yet; however prior studies have shown to improve functional status and in improving quality of life, and geriatric tools predicted survival and hospital utilization among older adults with hematologic malignancies (41).

The European VIVIFRAIL physical exercise prescription model that will be adopted in this study, and which has been developed by experts worldwide in the field of physical activity and frailty (and who are part of the research team), we consider being a milestone in this regard (42).

For the first time, significant progress has been made in the aforementioned individualized physical exercise prescription and its application to a population as vulnerable as the frail and cognitively impaired older adult. We bet that it can represent a significant advance in improving the functional capacity of these patients and that, as the World Health Organization says, it should be the objective of the health systems that care for these people. Our model is parallel and analogous to that posed by the concept of personalized medicine, since it individualizes the specific recommendations on exercise doses (intensity, volume, frequency) as is done with other medications.

The recruitment of this type of patient is a major challenge given the obvious differences compared to patients traditionally recruited in geriatric departments, with additional aspects beyond the purely medical issues, such as psychological aspects, the appropriate time to start complementary treatments or the interaction with baseline treatments such as chemotherapy when prioritizing the optimization of pharmacological and non-pharmacological therapies. All these factors will provide evidence that will help to update clinical guidelines related to this type of intervention in the future. At the present time, given recruitment of one-third of the initial estimate, we would have a power of almost 50% to detect as statistically significant a difference of 1 SPPB between groups.

If our hypothesis is correct, it opens the way to the modification of the hospitalization system and the current oncogeriatric management of patients with medical conditions. If we modify the current guidelines, it is likely that older adults after admission will present lower levels of functional and cognitive deterioration, predictably a better quality of life and lower consumption of health resources (lower readmissions, less institutionalization, among others).

## Conclusions

As previously stated, we anticipate that the results obtained by this study will inform future guidelines on the management of function and cognition in oncogeriatric hospitalized patients, highlighting the importance of including a physical exercise program for reverting the deterioration that frequently occurs in these patients.

## Availability of data and materials:

The datasets used and/or analyzed during the current study will be available from the corresponding author on reasonable request.

## Electronic Supplementary Material

Table 1: Baseline characteristics of the patients
