# Supplementary material for: Tailored Prevention of Functional Decline through a Multicomponent Exercise Program in Hospitalized Oncogeriatric Patients: Study Protocol for a Randomized Clinical Trial
Source: J Nutr Health Aging. 2023 Oct 5;27(10):911–8. doi: 10.1007/s12603-023-1977-y (PMC12880503; doi:10.1007/s12603-023-1977-y)
Supplement: Supplementary file 1 — Table 1: Baseline characteristics of the patients [file mmc1.docx]

Table 1: Baseline characteristics of the patients

| **Variable^1^** | **Control (n=30)** | **Intervention (n=28)** |
| --- | --- | --- |
| Age | 74.4 (5.2) | 74.4 (5.3) |
| Sex Men, n (%) | 18 (60.0%) | 17 (60.7%) |
| Living alone, n (%) | 6 (20.0%) | 4 (15.4%) |
| BMI | 25.7 (4.1) | 27.1 (5.8) |
| SPPB | 9.0 (2.5) | 7.9 (3.0) |
| GDS | 2.4 (1.7) | 2.4 (2.4) |
| Handgrip (Kg) | 26.2 (8.4) | 23.5 (7.1) |
| TMT-Part A (sec) | 60.5 (24.7) | 69.1 (40.0) |
| GVT 6m (sec) | 8.3 (3.6) | 9.6 (6.2) |
| Dual task GVT (n) | 10.7 (4.1) | 10.4 (2.7) |
| 1RM leg press (Kg) | 80.3 (28.9) | 69.1 (28.0) |
| 1RM knee extension (Kg) | 56.9 (22.1) | 44.0 (15.1) |
| 1RM bench press (Kg) | 20.4 (9.8) | 19.1 (10.0) |

^1^mean (sd)

BMI: Body mass index

SPPB: Short Performance Physical Battery

GDS: Glasgow Depression Scale

TMT: Trail Making Test

GVT: Gait Velocity Test

1RM: 1-repetition maximum
